# Supplementary material for: The autism- and schizophrenia-associated protein CYFIP1 regulates bilateral brain connectivity and behaviour
Source: Nat Commun. 2019 Aug 1;10:3454. doi: 10.1038/s41467-019-11203-y (PMC6672001; doi:10.1038/s41467-019-11203-y)
Supplement: Supplementary file 1 — Supplementary Information [file 41467_2019_11203_MOESM1_ESM.pdf]

Supplementary Information

The autism- and schizophrenia-associated protein CYFIP1 regulates bilateral brain connectivity and behaviour

Domínguez-Iturza et al.

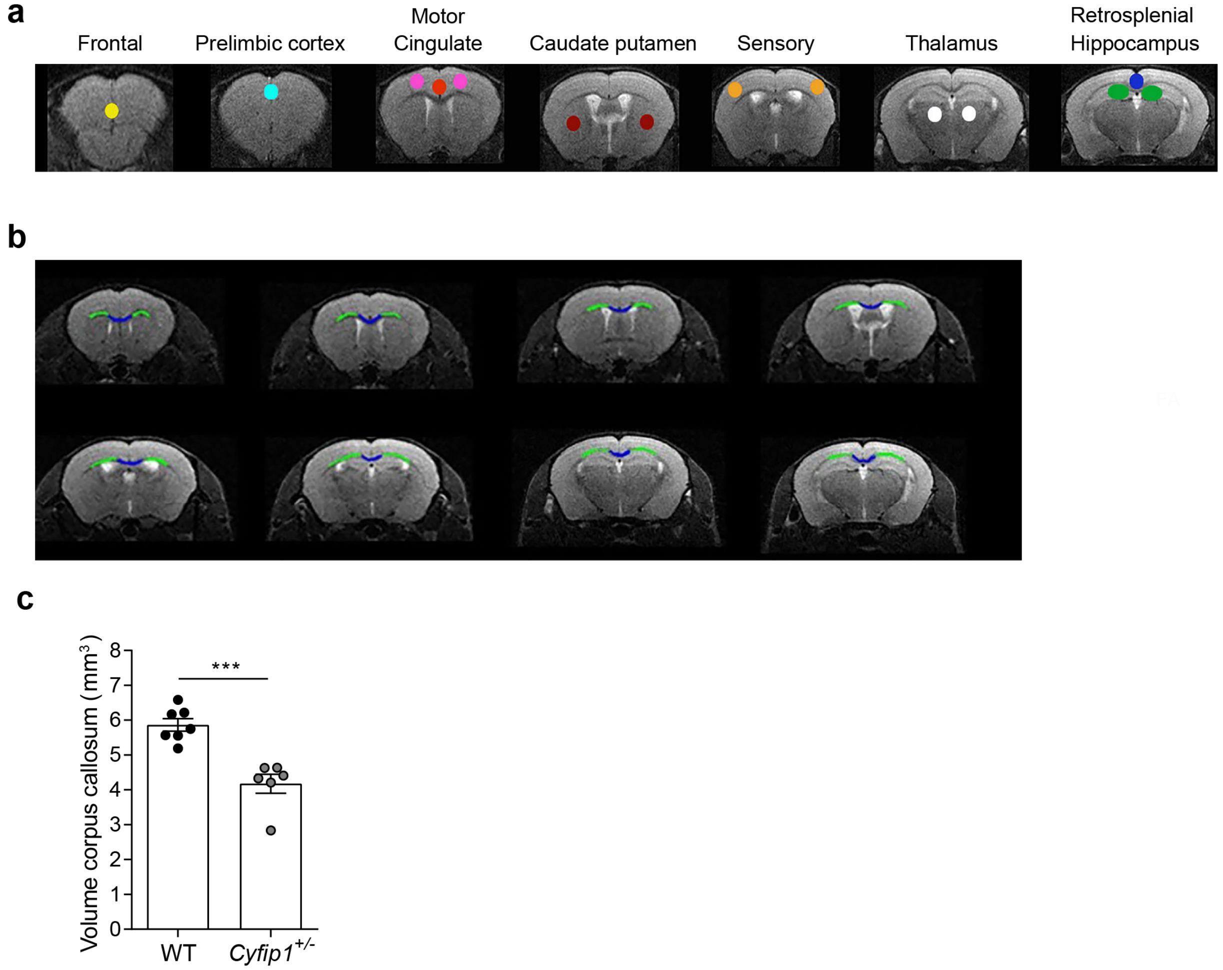

Supplementary Fig. 1 Supporting information concerning the MRI measurements. **a** Definition of the regions of interest (ROIs) used for the rsfMRI. Axial T2 weighted anatomical images and the selected seed (ROIs) for rsfMRI analysis. Yellow = orbitofrontal cortex, OFC; cyan = prelimbic cortex, PLC; red = cingulate cortex, Cg; fuchsia = motor cortex, MC; brown = caudate putamen, Cpu; orange = somatosensory cortex, SS; white = thalamus, T; blue = retrosplenial cortex, Resp; green = hippocampus, HC. **b** ROIs used in the DTI quantification. Axial T2 weighted anatomical images with delineation of the medial (blue) and lateral (green) corpus callosum region of interest (ROI) used for DTI analysis. **c** Volumetric measure of the corpus callosum. Shown is the volume of the ROIs delineated in **b** (n = 7 WT and n = 6 *Cyfip1*$^{+/-}$ mice) (mean ± SEM, two-tailed t-test, ***p = 0.0003).

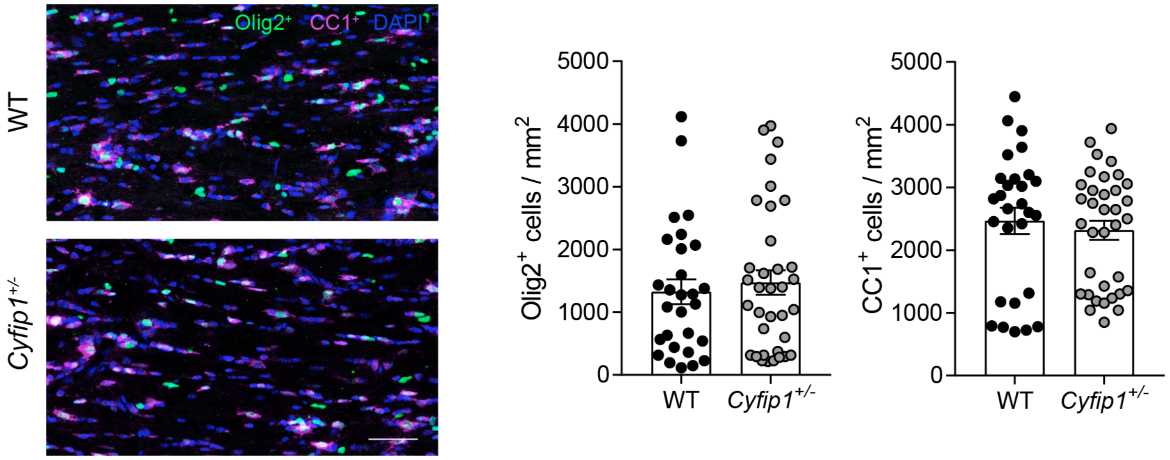

Supplementary Fig. 2 The number of oligodendrocytes in the corpus callosum does not change in adult *Cyfip1*$^{+/-}$ mice. Left, representative confocal images from the corpus callosum of WT and *Cyfip1*$^{+/-}$ mice at P60, stained for Olig2 (marker for immature cells of the oligodendrocyte lineage) and CC-1 (marker for mature myelinating oligodendrocytes) (scale bar 50μm). Right, quantification of the number of Olig2^{+} and CC1^{+} cells in WT and *Cyfip1*$^{+/-}$ mice (n = 28 and n = 35 slices from n = 7 and 9 mice, respectively) (mean ± SEM, not significant in two-tailed t-test).

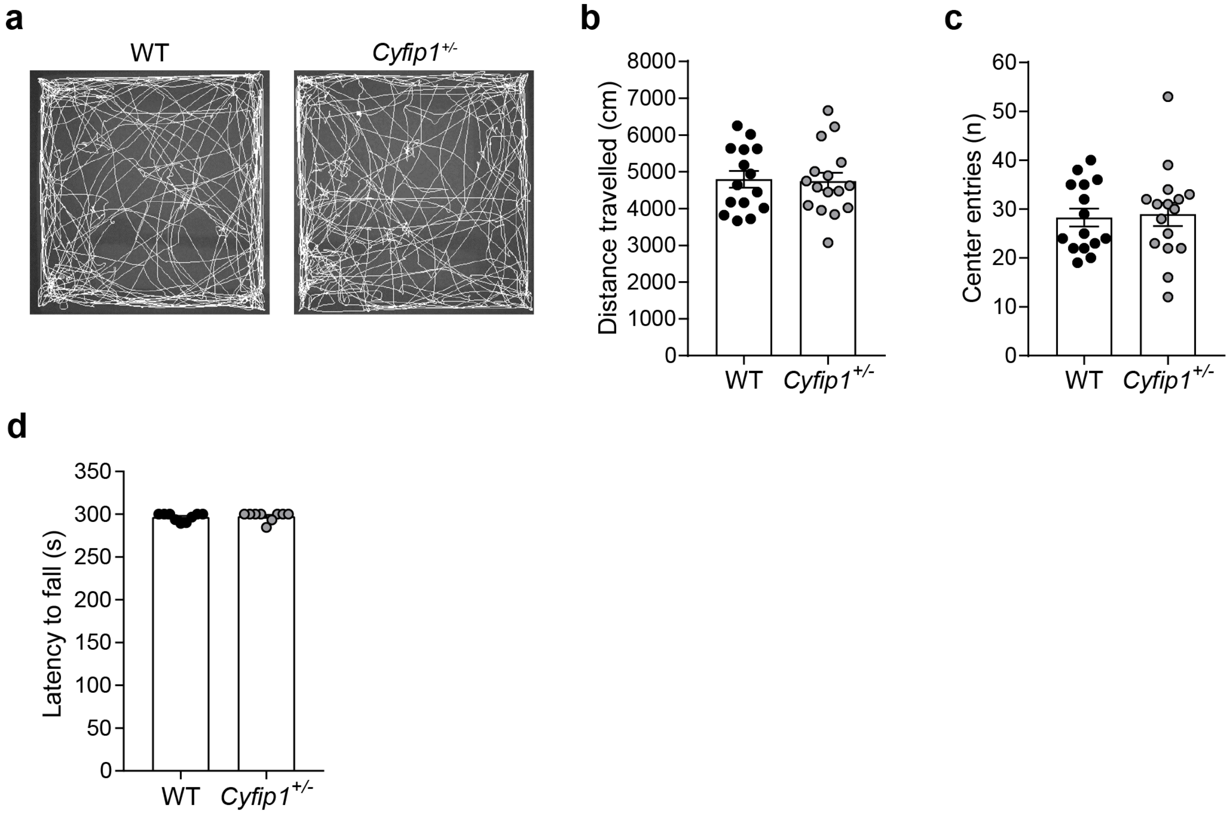

Supplementary Fig. 3 *Cyfip1*$^{+/-}$ mice have normal locomotion and muscle strength. **a** Representative movement tracks of WT and *Cyfip1*$^{+/-}$ mice in the open field. **b** and **c** Open field parameters measured for WT and *Cyfip1*$^{+/-}$ mice; distance travelled and center entries over a 10-min time period, respectively (WT n = 15 and *Cyfip1*$^{+/-}$ n = 16 mice) (mean ± SEM; not significant in two-tailed t-test). **d** Latency to fall measured in the hanging wire test for WT and *Cyfip1*$^{+/-}$ mice (WT n = 9 and *Cyfip1*$^{+/-}$ n = 9 mice) (mean ± SEM; not significant in two-tailed t-test).

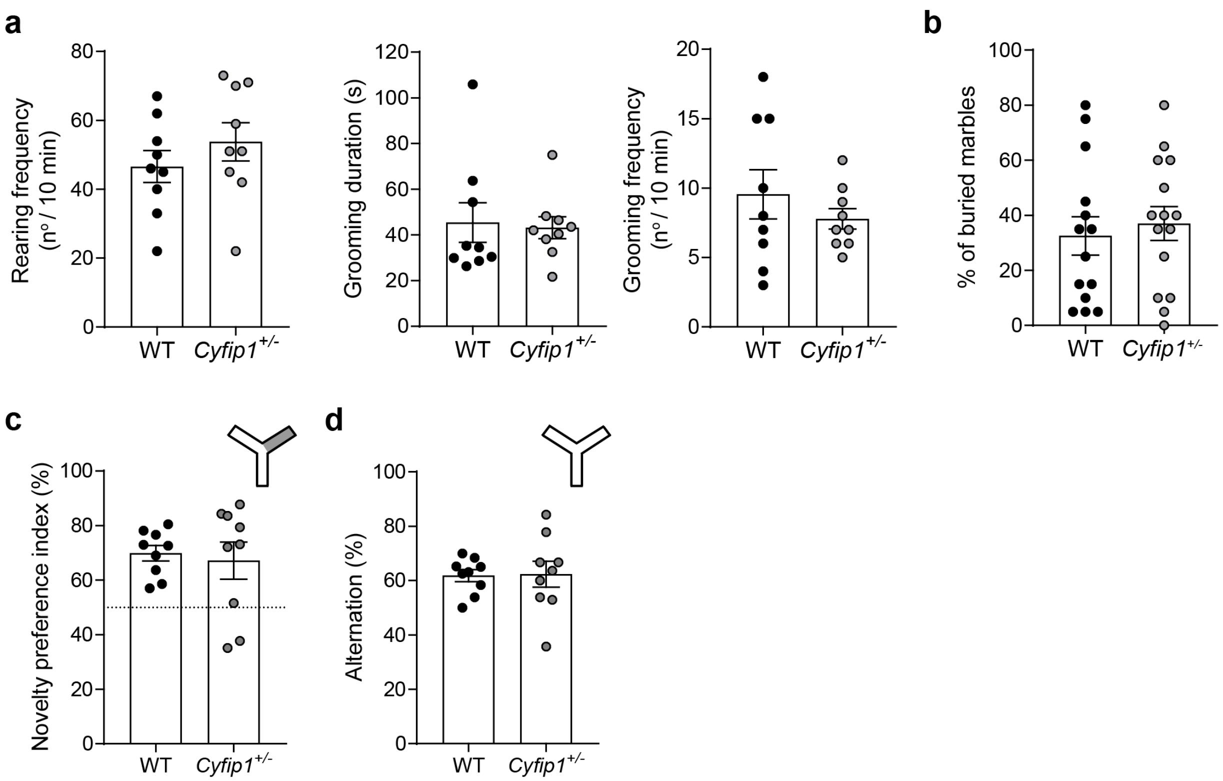

Supplementary Fig. 4 *Cyfip1*$^{+/-}$ mice have normal repetitive behaviours and working memory. **a** From left to right, rearing frequency, grooming duration and grooming frequency measured over a 10-min time period in WT and *Cyfip1*$^{+/-}$ mice (WT n = 9 and *Cyfip1*$^{+/-}$ n = 9 mice) (mean ± SEM; not significant in two-tailed t-test). **b** Percentage of buried marbles in WT and *Cyfip1*$^{+/-}$ mice (WT n = 14 and *Cyfip1*$^{+/-}$ n = 15 mice) (mean ± SEM; not significant in two-tailed t-test). **c** Estimate of working memory performance measured by the novelty preference index using the forced alternation paradigm of the Y-maze in WT and *Cyfip1*$^{+/-}$ mice (WT n = 9 and *Cyfip1*$^{+/-}$ n = 9 mice) (mean ± SEM; not significant in two-tailed t-test). **d** Spontaneous alternation measured in the Y-maze (WT n=9 and *Cyfip1*$^{+/-}$ n=9 mice) (mean ± SEM; not significant in two-tailed t-test).

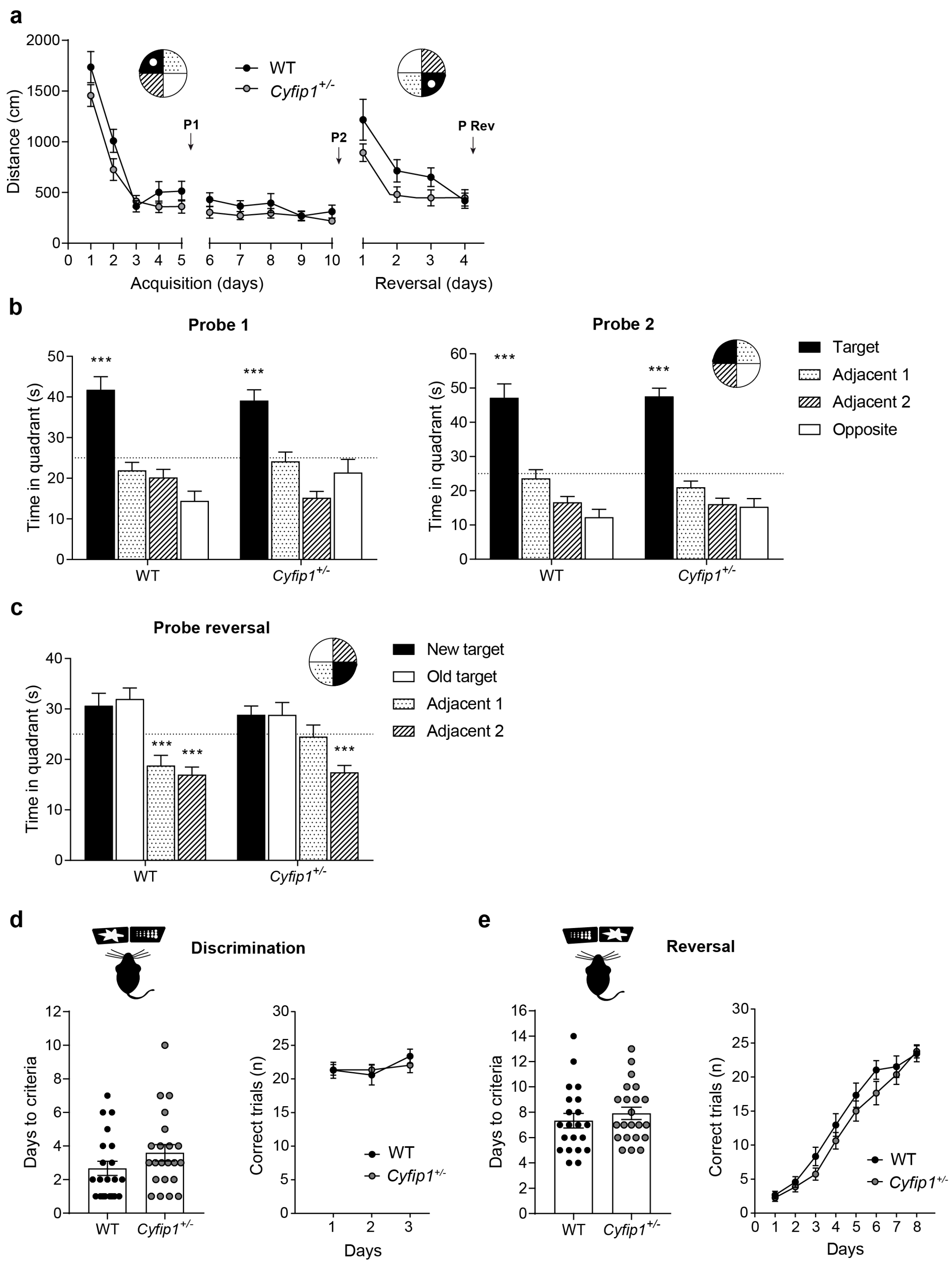
a
Distance (cm)
WT
Cyfip1+/-
P1
P2
P Rev
Acquisition (days)
Reversal (days)
b
Probe 1
Probe 2
Time in quadrant (s)
***
WT
Cyfip1+/-
Target
Adjacent 1
Adjacent 2
Opposite
c
Probe reversal
New target
Old target
Adjacent 1
Adjacent 2
d
Discrimination
Days to criteria
Correct trials (n)
Days
e
Reversal

Supplementary Fig. 5 *Cyfip1*$^{+/-}$ mice have no major cognitive flexibility defects. **a** Distance travelled to find the platform in the Morris water maze (MWM) for each acquisition and reversal day in WT and *Cyfip1*$^{+/-}$ mice. Probe trials were performed after 5 and 10 days of acquisition (Probe 1, P1, and Probe 2, P2, respectively) and the last day of reversal (Probe reversal) (WT n = 15 and *Cyfip1*$^{+/-}$ n = 18 mice) (mean ± SEM; no effect of genotype in Two-way Repeated-Measures ANOVA). **b** Left, time spent in each quadrant during Probe 1 (WT n = 15 and *Cyfip1*$^{+/-}$ n = 18 mice) (mean ± SEM; Two-way ANOVA, $F_{(3,124)} = 36.37$ ***$p < 0.0001$ for the target quadrant over the others for both WT and *Cyfip1*$^{+/-}$ mice). Right, time spent in each quadrant during Probe 2 (WT n = 15 and *Cyfip1*$^{+/-}$ n = 18 mice) (mean ± SEM; Two-way ANOVA, $F_{(3,124)} = 80.45$, ***$p < 0.0001$ for the target quadrant over the others for both WT and *Cyfip1*$^{+/-}$ mice). **c** Time spent in each quadrant during Probe reversal (WT n = 15 and *Cyfip1*$^{+/-}$ n = 18 mice) (mean ± SEM; Two-way ANOVA, $F_{(3,124)} = 19.46$, WT: ***$p = 0.0005$ for target vs. adjacent 1 and ***$p < 0.0001$ for target vs. adjacent 2; *Cyfip1*$^{+/-}$: ***$p = 0.0002$ for target vs. adjacent 2). **d** Left, days to criteria (80% correct, 24 out of 30) during the discrimination training phase of the touchscreen visual discrimination task (WT n = 21 and *Cyfip1*$^{+/-}$ n = 22 mice) (mean ± SEM; not significant in two-tailed t-test). Right, correct trials at different days during the discrimination training phase (WT n = 21 and *Cyfip1*$^{+/-}$ n = 22 mice) (mean ± SEM; no effect of genotype in Two-way Repeated-Measures ANOVA). **e** Days to criteria (80% correct) during the reversal phase of the touchscreen visual discrimination task (WT n = 21 and *Cyfip1*$^{+/-}$ n = 22 mice) (mean ± SEM; not significant in two-tailed t-test). Right, correct trials at different days during the reversal phase (WT n = 21 and *Cyfip1*$^{+/-}$ n = 22 mice) (mean ± SEM; no effect of genotype in Two-way Repeated-Measures ANOVA).
