# Supplementary material for: The autism- and schizophrenia-associated protein CYFIP1 regulates bilateral brain connectivity and behaviour
Source: Nat Commun. 2019 Aug 1;10:3454. doi: 10.1038/s41467-019-11203-y (PMC6672001; doi:10.1038/s41467-019-11203-y)
Supplement: Supplementary file 3 — Reporting Summary [file 41467_2019_11203_MOESM3_ESM.pdf]

nature research

Corresponding author(s): Claudia Bagni

Last updated by author(s): Mar 7, 2019

Reporting Summary

Nature Research wishes to improve the reproducibility of the work that we publish. This form provides structure for consistency and transparency in reporting. For further information on Nature Research policies, seeAuthors & Referees and theEditorial Policy Checklist .

Statistics

For all statistical analyses, confirm that the following items are present in the figure legend, table legend, main text, or Methods section.

n/a	Confirmed	
☐	☒	The exact sample size (*n*) for each experimental group/condition, given as a discrete number and unit of measurement
☐	☒	A statement on whether measurements were taken from distinct samples or whether the same sample was measured repeatedly
☐	☒	The statistical test(s) used AND whether they are one- or two-sided<br>*Only common tests should be described solely by name; describe more complex techniques in the Methods section.*
☐	☒	A description of all covariates tested
☐	☒	A description of any assumptions or corrections, such as tests of normality and adjustment for multiple comparisons
☐	☒	A full description of the statistical parameters including central tendency (e.g. means) or other basic estimates (e.g. regression coefficient) AND variation (e.g. standard deviation) or associated estimates of uncertainty (e.g. confidence intervals)
☐	☒	For null hypothesis testing, the test statistic (e.g. *F*, *t*, *r*) with confidence intervals, effect sizes, degrees of freedom and *P* value noted<br>*Give P values as exact values whenever suitable.*
☒	☐	For Bayesian analysis, information on the choice of priors and Markov chain Monte Carlo settings
☒	☐	For hierarchical and complex designs, identification of the appropriate level for tests and full reporting of outcomes
☒	☐	Estimates of effect sizes (e.g. Cohen's *d*, Pearson's *r*), indicating how they were calculated

Our web collection on statistics for biologists contains articles on many of the points above.

Software and code

Policy information about availability of computer code

Data collection	Detailed description can be found in the material and methods section
Data analysis	Detailed description can be found in the material and methods section

For manuscripts utilizing custom algorithms or software that are central to the research but not yet described in published literature, software must be made available to editors/reviewers. We strongly encourage code deposition in a community repository (e.g. GitHub). See the Nature Research guidelines for submitting code & software for further information.

Data

Policy information about availability of data

All manuscripts must include a data availability statement. This statement should provide the following information, where applicable:

- Accession codes, unique identifiers, or web links for publicly available datasets
- A list of figures that have associated raw data
- A description of any restrictions on data availability

All relevant data are available from the authors upon request

Field-specific reporting

Please select the one below that is the best fit for your research. If you are not sure, read the appropriate sections before making your selection.

☒ Life sciences ☐ Behavioural & social sciences ☐ Ecological, evolutionary & environmental sciences

For a reference copy of the document with all sections, see nature.com/documents/nr-reporting-summary-flat.pdf

Life sciences study design

All studies must disclose on these points even when the disclosure is negative.

Sample size	We chose the sample size based on the literature in the field and preliminary data from our laboratory.
Data exclusions	Outliers as defined by the 1.5*IQR formula were removed (this applied only to one sample in Figure 7b).
Replication	All experiments were performed from several independent biological samples across different days. Some of the experiments were performed and/or analyzed by independent researchers. Attempts for replication were all successful.
Randomization	No randomization was required for our experiments.
Blinding	For most of the experiments the investigators were blind to the genotype during data collecting and analysis.

Reporting for specific materials, systems and methods

We require information from authors about some types of materials, experimental systems and methods used in many studies. Here, indicate whether each material, system or method listed is relevant to your study. If you are not sure if a list item applies to your research, read the appropriate section before selecting a response.

Materials & experimental systems

n/a	Involved in the study	
☐	☒	Antibodies
☒	☐	Eukaryotic cell lines
☒	☐	Palaeontology
☐	☒	Animals and other organisms
☒	☐	Human research participants
☒	☐	Clinical data

Methods

n/a	Involved in the study	
☒	☐	ChIP-seq
☒	☐	Flow cytometry
☐	☒	MRI-based neuroimaging

Antibodies

Antibodies used	rabbit anti-Olig2 1:10000, AB9610 Millipore; mouse anti-CC-1 1:200, Sigma OP80
Validation	Antibodies used have been previously published by other researchers (see manufactures web page for more information)

Animals and other organisms

Policy information about studies involving animals; ARRIVE guidelines recommended for reporting animal research

Laboratory animals	Detailed description can be found in the material and methods section
Wild animals	Not applicable
Field-collected samples	Not applicable
Ethics oversight	Detailed description can be found in the material and methods section

Note that full information on the approval of the study protocol must also be provided in the manuscript.

Magnetic resonance imaging

Experimental design

Design type	resting-state functional Magnetic Resonance Imaging (rsfMRI) and Diffusion Tensor Imaging (DTI)
Design specifications	rsfMRI: 150 repetitions of 2 seconds. DTI: repetition time 5,5s. Total scan time, including the acquisition of anatomical images < 1,5h/mouse
Behavioral performance measures	Not applicable

Acquisition

Imaging type(s)	functional and diffusion
Field strength	9.4T for rsfMRI and 7T for DTI
Sequence & imaging parameters	rsfMRI: gradient echo EPI, repetition time 2000 ms, echo time 15 ms, 16 slices of 0.5 mm, 150 repetitions, field-of-view (20 x 20) mm² and matrix size (128 x 64).<br>DTI:two shot spin echo EPI, Repetition time 5500 ms, echo time 23.23 ms, 18 slices of 0.5 mm, b=800 s/mm², 60 DW direction, field of view (20 x 20) mm² and matrix size (96 x96).
Area of acquisition	The entire cerebrum was imaged, between the olfactory bulb and the cerebellum

Diffusion MRI ☒ Used ☐ Not used

Parameters: b=800 s/mm², 60 DW directions, no cardiac gating

Preprocessing

Preprocessing software	SPM8 software (Statistical Parametric Mapping, http://www.fil.ion.ucl.ac.uk)<br>realignment:least-squares approach and a 6-parameter (rigid body) spatial transformation. For the analyses of the rsfMRI data, motion parameters resulting from the realignment were included as covariates to correct for possible movement that occurred during the scanning procedure<br>smoothing: twice the voxel size (0.31 X 0.62 mm²)
Normalization	All datasets were normalized to a study-specific EPI template. The normalization steps consisted of a global 12-parameter affine transformation followed by the estimation of the nonlinear deformations
Normalization template	All datasets were normalized to a study-specific EPI template
Noise and artifact removal	For the analyses of the rsfMRI data, motion parameters resulting from the realignment were included as covariates to correct for possible movement that occurred during the scanning procedure
Volume censoring	Datasets that showed movement of > voxel were discarded. Otherwise movement parameters were used as covariates in analyses

Statistical modeling & inference

Model type and settings	T-tests, p<0.05, FDR<br>Two-way ANOVA, p<0.05, Holm-Sidak
Effect(s) tested	T-test: group analysis<br>Two-way ANOVA: difference between wild-types and Cyfip1+/-

Specify type of analysis: ☐ Whole brain ☐ ROI-based ☒ Both

Anatomical location(s): anatomical regions were delineated manually based on the Franklin and Paxinos anatomical mouse brain atlas

Statistic type for inference (See Eklund et al. 2016)	voxel-wise
Correction	FDR

Models & analysis

n/a	Involved in the study
☐	☒ Functional and/or effective connectivity
☒	☐ Graph analysis
☒	☐ Multivariate modeling or predictive analysis

Functional and/or effective connectivity: Pearson correlations
